# Effects of Piracetam and Hyperbaric Oxygen on Histopathological and Circulating Biochemical Responses Following Sciatic Nerve Crush Injury in Rats

**DOI:** 10.3390/medicina62101904

**Published:** 2026-10-01

**Authors:** Umut Caner Canoğlu, Ilknur Suidiye Yorulmaz, Mehmet Gamsızkan, Abdulkadir İskender, Merve Alpay, Mehmet Ali Sungur, Mehrdad Sheikhvatan

**Affiliations:** 1 Anesthesiology and Reanimation Department, Istanbul Okan University, Istanbul 34959, Turkey; 2 Anesthesiology and Reanimation Department, Duzce University, Duzce 81620, Turkey; 3 Pathology Department, Duzce University, Duzce 81620, Turkey; 4 Anesthesiology and Reanimation Department, Medical Science University, Bursa 16000, Turkey; 5 Biochemistry Department, Duzce University, Duzce 81620, Turkey; 6 Biostatistics Department, Duzce University, Duzce 81620, Turkey; 7 Department of Medical Biology and Genetics, Istanbul Okan University, Istanbul 34959, Turkey

**Keywords:** piracetam, hyperbaric oxygen, nerve injury, animal model, regeneration

## Abstract

*Background and Objectives*: Peripheral nerve injury initiates a series of degenerative, inflammatory, and reparative responses. Piracetam and hyperbaric oxygen (HBO) have been proposed as potential therapeutic interventions; however, their effects during the early phase following sciatic nerve injury require further investigation. This study evaluated early histopathological and circulating biochemical responses to piracetam and HBO in a rat model of sciatic nerve injury. *Materials and Methods*: Forty male Wistar Albino rats were randomly allocated to four groups following experimental sciatic nerve injury: an untreated injury control group (Group K), a hyperbaric oxygen therapy group (Group HBO), an intraperitoneal piracetam group (Group P), and a combined hyperbaric oxygen plus piracetam group (Group HBO + P). After 14 days, the rats were euthanised, and sciatic nerve tissues and blood samples were collected for histopathological and biochemical analyses. *Results*: No statistically significant differences were observed among the four groups with respect to axonal continuity, capillary formation, or myelin sheath regeneration. Fibroblast density was increased in Group P compared with Group K (*p* = 0.025), while no significant differences were detected among the remaining groups. Inflammatory cell counts were higher in Group P than in Group K (*p* = 0.037) and elevated in Group HBO + P compared with Group K (*p* = 0.014). Hypoxia-inducible factor-1 alpha (HIF-1α) expression was highest in Group K. Additionally, neuron-specific enolase levels were significantly increased in Group P compared with Group K (*p* = 0.006). *Conclusions*: Under the conditions of this 14-day experimental study, piracetam and HBO were associated with differences in selected circulating biochemical parameters but did not produce statistically significant improvements in the assessed histopathological indices. The findings were interpreted as early responses to treatment, and longer follow-up studies are needed to determine whether these changes translate into sustained axonal regeneration or functional recovery.

## 1. Introduction

Peripheral nerve injuries remain a significant clinical challenge due to their high incidence and the often-incomplete functional recovery that follows despite advances in microsurgical repair techniques [1]. Sciatic nerve injury, one of the most commonly studied experimental models of peripheral nerve damage, closely mimics human traumatic neuropathies and provides a reliable platform for evaluating therapeutic interventions aimed at enhancing nerve regeneration and functional recovery [2,3]. Although spontaneous regeneration can occur in peripheral nerves, the process is slow and frequently insufficient, resulting in persistent motor and sensory deficits [4]. Consequently, there is an ongoing need to identify adjuvant therapies that can accelerate nerve repair and improve functional outcomes [5].

The pathophysiology of peripheral nerve injury involves a complex cascade of events, including axonal degeneration, Schwann cell dysfunction, inflammatory responses, oxidative stress, and impaired microcirculation at the injury site [6,7]. These mechanisms collectively hinder axonal regrowth and remyelination [7]. Pharmacological agents with neuroprotective and neurotrophic properties, as well as physical modalities that enhance tissue oxygenation and cellular metabolism, have therefore attracted increasing interest as potential strategies to support nerve regeneration [8].

Sciatic nerve crush injury is an experimental model used to investigate peripheral nerve damage and the biological responses associated with nerve repair. Histopathological examination and circulating biochemical markers may provide information about tissue injury, inflammation, and treatment-associated biological responses. However, these outcomes do not independently establish the restoration of motor or sensory function. Objective functional assessments, such as walking-track analysis, the Sciatic Functional Index, muscle-strength testing, sensory evaluation, or electrophysiological examination, are required to quantify functional recovery.

Piracetam, a cyclic derivative of gamma-aminobutyric acid (GABA), is widely recognized for its neuroprotective, nootropic, and rheological effects [9]. Experimental studies have demonstrated that piracetam improves neuronal metabolism, enhances microcirculatory blood flow, reduces oxidative stress, and stabilizes neuronal membranes [10,11]. In the context of nerve injury, these properties suggest a potential role for piracetam in promoting axonal regeneration and preserving neural integrity, yet evidence regarding its efficacy in peripheral nerve repair remains limited and warrants further investigation [12].

Hyperbaric oxygen (HBO) therapy is another therapeutic modality that has gained attention for its beneficial effects in neural tissue repair [13]. By increasing the partial pressure of oxygen in plasma, HBO enhances oxygen delivery to hypoxic tissues, reduces edema, modulates inflammatory responses, and stimulates angiogenesis [14]. Several experimental and clinical studies have reported that HBO therapy can improve nerve regeneration and functional recovery following peripheral nerve injury [15]; however, the optimal application protocols and its potential synergistic effects with pharmacological agents have not been fully elucidated.

Peripheral nerve crush injury may induce local edema, impaired microcirculation, inflammation, and increased metabolic demands, potentially compromising oxygen availability in the injured nerve tissue. Although tissue hypoxia was not directly measured in the present study, these injury-related changes provide a biological rationale for investigating hyperbaric oxygen (HBO) therapy. HBO may increase tissue oxygen availability and potentially support nerve repair through improved oxygenation, modulation of edema and inflammation, and preservation of cellular integrity. Previous experimental studies have reported variable effects of HBO on peripheral nerve regeneration, including potential improvements in axonal growth and tissue recovery. However, these findings remain inconsistent and may depend on the injury model and treatment protocol. Therefore, HBO was investigated in the present study as a potential adjunctive intervention for sciatic nerve repair, rather than as an established regenerative treatment [16].

Given the distinct yet potentially complementary mechanisms of piracetam and hyperbaric oxygen therapy, their combined or comparative effects on peripheral nerve regeneration merit systematic evaluation. Therefore, the aim of this study was to investigate the effects of piracetam and hyperbaric oxygen therapy on histopathological findings and circulating biochemical responses in rats following experimentally induced sciatic nerve crush injury. By assessing functional, histopathological, and biochemical outcomes, this study seeks to contribute to the growing body of evidence on adjuvant therapies that may enhance peripheral nerve repair and inform future translational approaches.

## 2. Materials and Methods

### 2.1. Study Plan

This study used 40 male Wistar-Albino rats (Düzce University, Düzce Faculty of Medicine, Experimental Animal Application and Research Center). Prior to the study, the necessary permissions were obtained from the Düzce University Düzce Faculty of Medicine Experimental Animal Local Ethics Committee (1 October 2020). The study was conducted in the Düzce University Düzce Faculty of Medicine Experimental Animal Application and Research Center Laboratory. Laboratory animal care principles were followed in this study. The average age of the rats included in the study was 2.5 months (2–3 months), and their average weight was 250 g (200–300 g). The animals were randomly divided into 4 groups, with 10 rats in each cage, and monitored in the laboratory environment for 1 week preoperatively. During the study, the rats were given unlimited tap water (ad libitum) and standard rodent feed. The animals were monitored in cages in a room with controlled temperature (23–25 °C), a 12/12 h light/dark cycle, and 50–60% humidity. The animals used in the study were monitored in their cages for approximately one week without any procedures after being brought to the laboratory center. On the day of the study, the rats were brought to the research laboratory and weighed. Anesthesia was achieved by administering intramuscular ketamine hydrochloride 90 mg/kg (Ketalar^®^, Eczacıbaşı İlaç Sanayi ve Ticaret A.Ş., Istanbul, Turkey) and intramuscular 6 mg/kg xylazine (Rompun^®^, Bayer Türk Kimya Sanayi, Istanbul, Turkey) to the rats. The individual rat was considered the experimental unit for the histopathological and biochemical analyses. The 40 animals were allocated to four experimental groups, with 10 rats per group. In the housing arrangement, each group consisted of two cages, with five rats housed in each cage. Animals were distributed among cages within their assigned treatment groups, and cage locations were periodically rotated or balanced to minimize potential environmental effects. The primary statistical analyses were conducted at the individual-animal level.

### 2.2. Sciatic Nerve Injury Model

**Figure 1:**
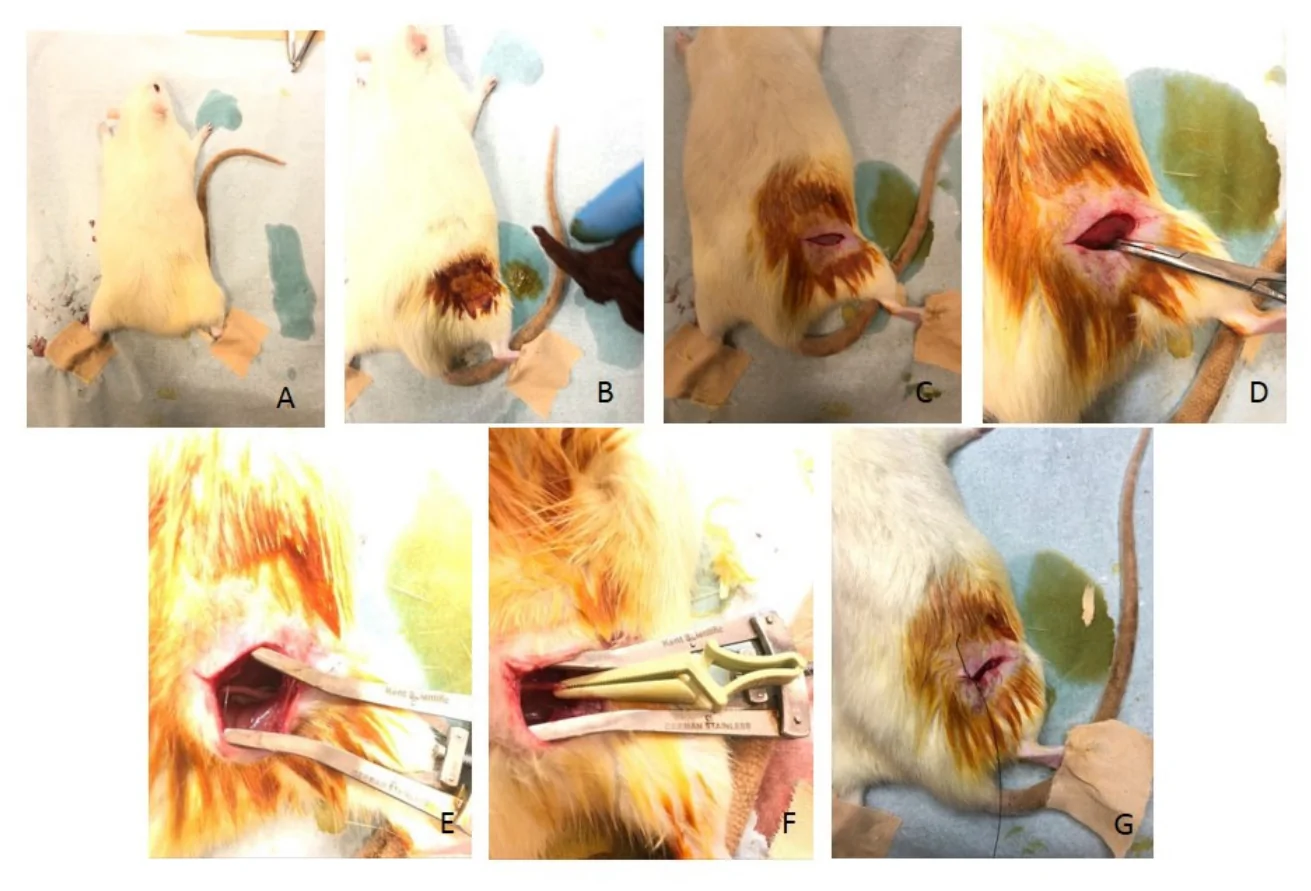
The anesthetized rat is placed in the surgical position (**A**), Cleaning the skin with an antiseptic solution (**B**), Making a skin incision (**C**), Dissection of subcutaneous and muscle tissue (**D**), Location of the sciatic nerve (**E**), Sciatic nerve compression with clipping (**F**), Covering the skin (**G**).

Following anesthesia, each rat was placed in the prone position, and the right gluteal region was prepared using a 10% povidone–iodine solution. A longitudinal skin incision approximately 2–3 cm in length was made in the gluteal region, followed by blunt dissection of the subcutaneous tissue and gluteal muscles to expose the right sciatic nerve. A segment of approximately 2 cm of the sciatic nerve was carefully isolated. The nerve injury was induced using a small, atraumatic bulldog vascular clamp (Atraumatic Bulldog Clamp, Fine Tip, 12 mm jaw—Aesculap, Tuttlingen, Germany, model FB480R). The clip was applied to the exposed sciatic nerve at the midportion of the isolated segment, approximately 5 mm distal to the proximal end of the exposed nerve segment. The clip was maintained in position for approximately 30 s during each application. The procedure was repeated three times at the same anatomical site, with an interval of approximately 30 s between applications. The clip was applied perpendicular to the longitudinal axis of the nerve, and consistent positioning was maintained by using the same anatomical segment and surgical approach in all animals. The same injury protocol was applied to all animals assigned to the experimental injury groups. After completion of the injury procedure, the clip was removed, and the surgical field was inspected for visible bleeding or other complications. The muscle layer was approximated using absorbable 4-0 surgical sutures, and the skin incision was closed using interrupted 4-0 nylon sutures. The animals were then transferred to the recovery area and monitored according to the approved animal-care protocol. (Figure 1). Following sciatic nerve injury, foot drop was observed in the affected extremity. This observation was recorded as a clinical sign associated with the injury. Because no validated motor or sensory functional assessment was performed, foot drop observations were not used to calculate a quantitative functional recovery score.

### 2.3. Study Groups

Before the intervention, the animals were assigned to four experimental groups using a computer-generated random allocation sequence. The sequence was generated by a researcher who was not involved in treatment administration or outcome assessment. No stratification or restriction was used, and equal allocation was planned, with 10 rats assigned to each group. Allocation concealment was maintained until group assignment, after which the assigned intervention was administered according to the group designation. Blinding procedures were considered separately for treatment administration, sample processing, histopathological assessment, and statistical analysis. Personnel involved in surgery and treatment administration were aware of group assignments because this information was required to administer the designated interventions. For the histopathological assessment, tissue samples were identified using coded labels, and the evaluator was blinded to treatment allocation during microscopic examination and semiquantitative scoring. Axonal continuity, fibroblast count, myelin sheath thickness, inflammatory cell infiltration, and capillary density were assessed using the predefined scoring system. Biochemical analyses were performed using coded samples, where applicable, to minimize the potential influence of knowledge of treatment allocation. Statistical analyses were conducted using coded group identifiers, with group identities unmasked after the primary analyses had been completed. Prophylactic antibiotics were administered to all groups after surgery. No rats died in any group after the operation. No wound infection was observed in any of the rats during follow-up. The control group (n = 10) was named Group K, the hyperbaric oxygen group (n = 10) was named Group HBO, the piracetam group (n = 10) was named Group P, and the group given both HBO and piracetam (n = 10) was named Group HBO + P. Group K (untreated injury control group): Sciatic nerve injury was induced, but no specific therapeutic intervention was administered. The animals received standard postoperative care throughout the 14-day follow-up period. At the end of the study, euthanasia was performed under anesthesia, and macroscopic and histological evaluations were conducted. Group K served as the untreated injury control group for comparisons with the HBO-, piracetam-, and combined-treatment groups. Group HBO (HBO treated group): HBO treatment was given for 1 h a day for 14 days under a pressure of 2.5 ATA. At the end of the 14th day, euthanasia was performed by cervical dislocation under anesthesia/tranquilizers, and macroscopic and histological evaluations were carried out. Group P (Piracetam-treated group): 250 mg/kg piracetam was administered intraperitoneally in two equal doses twice daily. At the end of the 14th day, euthanasia was performed by cervical dislocation under anesthesia/tranquilizers, and macroscopic and histological evaluations were carried out. Group HBO + P (Piracetam and HBO-treated group): 250 mg/kg piracetam was administered intraperitoneally in two equal doses twice daily. HBO treatment was given for 1 h a day at 2.5 ATA pressure for 14 days. At the end of the 14th day, euthanasia was performed by cervical dislocation under anesthesia/tranquilizers, and macroscopic and histological evaluations were carried out. Blood samples were taken from the hearts of all groups before sacrifice. Blood samples were centrifuged to separate the plasma. The resulting samples were stored at −80 °C. Biochemical parameters such as NSE, S100B, and HIF-1α markers were analyzed.

### 2.4. Anesthesia and Perioperative Monitoring

Anesthesia was induced by intramuscular administration of ketamine hydrochloride (90 mg/kg; Ketalar^®^, Eczacıbaşı İlaç Sanayi ve Ticaret A.Ş., Istanbul, Turkey) and xylazine (6 mg/kg; Rompun^®^, Bayer Türk Kimya Sanayi, Istanbul, Turkey). The same anesthetic regimen was used for all animals undergoing the sciatic nerve injury procedure. The commercial product names, formulations, and manufacturers were harmonized as follows: Ketalar^®^ (ketamine hydrochloride, 50 mg/mL; Eczacıbaşı İlaç Sanayi ve Ticaret A.Ş., Istanbul, Turkey) and Rompun^®^ (xylazine hydrochloride, 20 mg/mL; Bayer Türk Kimya Sanayi, Istanbul, Turkey). These formulations were used consistently throughout the experimental procedures. Following administration of the anesthetic agents, anesthesia depth was assessed by evaluating the pedal withdrawal reflex, response to tactile stimulation, respiratory pattern, and muscle relaxation. The surgical procedure was initiated only after the absence of an appropriate pedal withdrawal response and confirmation of adequate anesthesia. Respiratory rate, mucous membrane color, and spontaneous movement were monitored throughout the procedure. Supplemental anesthesia was administered when necessary, based on the return of the pedal withdrawal reflex, purposeful movement, or other signs of inadequate anesthetic depth. The supplemental dose, timing, and maximum permitted number of booster injections were verified from the original experimental records. During anesthesia and surgery, the animals were placed on a temperature-controlled heating pad covered with a protective insulating layer, and body temperature was maintained at approximately 37 ± 1 °C. Body temperature was monitored using a rectal thermometer at regular intervals throughout the procedure. The heating device was adjusted as necessary to prevent perioperative hypothermia or overheating. After surgery, the animals were transferred to a warm recovery area and observed until the return of spontaneous movement and adequate recovery from anesthesia.

### 2.5. Hyperbaric Oxygen Therapy

Animals assigned to the HBO groups received one hyperbaric oxygen session per day for 14 consecutive days. Each session consisted of a 5 min compression period, exposure to 100% oxygen at 2.5 ATA for 60 min, and a 10 min decompression period. The chamber was flushed with 100% oxygen for 5 min before treatment. The total duration of each session was approximately 80 min. The treatment pressure of 2.5 ATA represents the absolute pressure maintained during the 60 min treatment phase. HBO-treated and control animals were handled concurrently. During the corresponding chamber procedures, animals remained in their home cages. Animals in the control group underwent sham chamber exposure with comparable handling and chamber-related procedures, including placement in the chamber and the corresponding exposure period, but without therapeutic hyperbaric oxygen administration. This procedure was used to minimize differences attributable to handling and chamber exposure between groups.

### 2.6. Piracetam Treatment

Rats assigned to Group P (piracetam-treated group) and Group HBO + P (combined piracetam and hyperbaric oxygen group) received piracetam (Nootropil^®^; UCB Pharma, Brussels, Belgium; 800 mg/5 mL oral solution) at a total daily dose of 250 mg/kg/day. The daily dose was divided into two equal intraperitoneal administrations of 125 mg/kg per injection, administered approximately 12 h apart. Treatment was continued for 14 consecutive days, beginning on the day of sciatic nerve injury. The same dosing regimen was used in both piracetam-treated groups. No complications related to piracetam administration were observed during the reported follow-up period.

### 2.7. Clinical Follow-Up and Evaluation

Clinically, right foot drop was observed in all rats in Group K, Group HBO, Group P, and Group HBO + P in which sciatic nerve damage was induced. Treatment with HBO and piracetam was administered for 14 days under standard care conditions. All rats in Groups K, HBO, P, and C were euthanised at the end of the 14th day, and their sciatic nerves were removed for histopathological examination.

### 2.8. Histopathological Examination

At the end of the 14-day treatment period, the animals were euthanized according to the approved experimental protocol, and the injured right sciatic nerves were carefully harvested for histopathological examination. The tissue samples were collected from the region of the experimentally induced nerve injury, including the lesion centre and adjacent nerve segments. The specimens were fixed in 10% neutral buffered formalin, processed using standard histological procedures, embedded in paraffin, and sectioned at a thickness of approximately 5 μm. Sections were stained with hematoxylin and eosin (H&E) for the evaluation of general tissue morphology and cellular changes. For each animal, three representative sections were selected for histopathological evaluation. The sections were obtained at intervals of approximately 100–200 μm to reduce the likelihood of evaluating the same microscopic region repeatedly. The sections were examined in the transverse orientation, and the lesion centre was assessed together with the adjacent proximal and distal nerve segments, where available. The primary assessment region was defined before microscopic evaluation. Histological evaluation included hematoxylin and eosin (H&E), toluidine blue, and Masson’s trichrome staining. H&E staining was used to evaluate general tissue morphology, including axonal organization, cellular changes, and inflammatory-cell infiltration. Toluidine blue staining was used to assess nerve-fibre and myelin-associated morphological features, whereas Masson’s trichrome staining was used to evaluate connective-tissue and collagen-associated changes. The specific interpretation of each stain was based on the histological structures identifiable in the evaluated sections. Immunohistochemical staining for neurofilament and CD34 was also reported in the original study protocol. Neurofilament staining was intended to evaluate neuronal or axonal structural features, whereas CD34 staining was intended to assess vascular-associated immunoreactivity. The immunohistochemical procedures were performed using the laboratory’s established protocol [17]. The histological and immunohistochemical preparations were evaluated in the Department of Pathology, Düzce University Faculty of Medicine. Representative histological images were obtained from the sciatic nerve samples of each experimental group and evaluated alongside the semi-quantitative histopathological findings. Images were presented with the corresponding staining method, magnification, and scale bars. The histopathological parameters were evaluated using a semiquantitative scoring system in which higher scores represented relatively more favorable tissue preservation or regeneration, whereas lower scores represented greater pathological alteration. The direction of scoring was specified separately for each parameter because the biological meaning of a higher score may differ between structural preservation, fibroblast density, inflammatory-cell infiltration, and capillary density. The operational definitions for scores 1, 2, and 3 are presented in Table 1. Five histopathological parameters were assessed: (1) axonal continuity, (2) fibroblast density, (3) myelin sheath preservation or regeneration, (4) inflammatory-cell infiltration, and (5) capillary density. Each parameter was graded using a three-point semiquantitative scale ranging from 1 to 3. A score of 1 represented a severe abnormality or limited preservation, a score of 2 represented an intermediate finding, and a score of 3 represented relatively preserved tissue morphology or a more pronounced regenerative response [18]. The specific operational criteria for each parameter are presented in Table 1. Neurofilament and CD34 staining findings were evaluated quantitatively [19]. The immunohistochemical findings were summarized at the animal level, where applicable, to avoid treating individual microscopic fields as independent experimental observations.

The scores for the microscopic fields were summarized to obtain a representative score for each parameter for each animal. The animal, rather than the individual microscopic field, was considered the experimental unit for statistical analysis. The final histopathological score was calculated by summing the individual parameter scores. Higher scores indicated relatively better structural preservation and more pronounced regeneration. Histopathological assessment was performed by one observer with verified professional qualifications and relevant experience. The observer was blinded to the treatment groups. The slides were coded by an investigator who did not participate in the microscopic assessment, and group identities were disclosed after completion of the scoring process.

### 2.9. Biochemical Analysis

All biochemical measurements were performed in the Medical Biochemistry Department Laboratory of Düzce University Faculty of Medicine by the same biochemistry specialist. Blood samples were collected from the heart of each rat immediately before euthanasia. The samples were centrifuged at 3000 rpm for 10 min to separate the serum. The serum fraction was carefully collected using a micropipette, transferred into Eppendorf tubes, and stored at −80 °C until biochemical analysis. Serum hypoxia-inducible factor-1 alpha (HIF-1α), S100 calcium-binding protein B (S100B), and neuron-specific enolase (NSE) concentrations were measured using commercially available rat enzyme-linked immunosorbent assay (ELISA) kits (USCN, Wuhan, China), according to the manufacturers’ instructions. The corresponding kit lot numbers were HIF-1α (L210213034), S100B (L20405260), and NSE (L210407794). For each assay, standards and serum samples were prepared according to the respective kit protocol. Following sample and standard preparation, the appropriate detection reagents were added according to the manufacturer’s instructions, with the specified incubation periods and washing steps. Detection reagent A was incubated at 37 °C for 1 h, followed by washing and addition of detection reagent B, which was incubated at 37 °C for 30 min. After five washing cycles, the substrate solution was added and incubated at 37 °C for 20 min. The reaction was subsequently terminated using the stop solution. Absorbance was measured at 450 nm using a BioTek Epoch microplate reader (BioTek Instruments, Winooski, VT, USA). Standard curves were generated according to the respective assay protocol, and sample concentrations were calculated using Gen5 software (version 5.2.1; BioTek Instruments, Winooski, VT, USA). The concentrations of HIF-1α, S100B, and NSE were reported using the measurement units specified in the corresponding manufacturer’s ELISA documentation. No polymerase chain reaction (PCR) procedure or PCR instrument was used for these biochemical measurements. The previous terminology referring to “PCR,” “PCR instrument,” and “calorimetric” measurement was therefore corrected to accurately describe the ELISA-based colorimetric assay and microplate-reader procedure.

### 2.10. Statistical Analysis

Statistical analyses were performed using IBM SPSS Statistics version 22.0. The individual rat was considered the experimental unit, and each rat contributed one observation per outcome unless otherwise specified. The data were screened for missing values, implausible observations, and potential outliers before analysis. No animal was treated as an independent replicate for outcomes measured from multiple sections or fields of the same animal. Continuous variables were summarized using the mean and standard deviation when approximately normally distributed and the median, interquartile range, and minimum–maximum values when distributional assumptions were not met. The distribution of continuous outcomes was assessed using graphical methods and the Shapiro–Wilk test. Homogeneity of variances was assessed using Levene’s test. The results of these assumption checks were used to guide the selection of the statistical methods. Body weight was analyzed separately from the terminal outcomes. The exact measurement time points are reported in the revised Results section. When repeated measurements were available, body weight was analyzed using a linear mixed-effects model with treatment group, time, and the group-by-time interaction as fixed effects and rat as a random effect. The covariance structure was selected according to model fit and the repeated-measures data structure. Baseline body weight was considered in the model where appropriate. The group-by-time interaction was used to assess whether changes in body weight over time differed among treatment groups. For continuous terminal outcomes, including serum biomarker concentrations, a one-way analysis of variance (ANOVA) was used when the assumptions of approximate normality and homogeneity of variances were adequately met. When the overall ANOVA was significant, pairwise comparisons were performed using Tukey’s honestly significant difference procedure, with the multiplicity adjustment specified in the statistical output. When the assumptions for parametric analysis were not met, the Kruskal–Wallis test was used, followed by Dunn’s pairwise comparisons with Bonferroni adjustment for multiple comparisons. The selected post hoc procedure and adjusted *p* values are reported in the corresponding tables. Histopathological variables were evaluated as ordinal semi-quantitative scores and were not treated as continuous measurements without justification. The primary analysis used the Kruskal–Wallis test to compare score distributions across the four groups, followed by adjusted pairwise Dunn tests when the omnibus test was statistically significant. The distribution of each score category was also presented using counts and percentages. If an ordinal regression model was used in the revised analysis, the model specification, proportional-odds assumption, estimated effect measures, and confidence intervals were reported. To reduce the risk of type I error associated with multiple outcomes and pairwise comparisons, the family of confirmatory outcomes and the multiple-comparison procedure were prespecified or reconstructed from the original analysis plan where possible. The manuscript distinguishes omnibus tests from post hoc pairwise comparisons and reports adjusted *p* values where available. Because the study evaluated several histopathological and biochemical outcomes, the findings are interpreted as exploratory unless a prespecified multiplicity-control strategy was applied. The exact correction method used for each family of comparisons is reported in the revised statistical analysis and results tables. For each relevant analysis, effect sizes and corresponding 95% confidence intervals will be reported where they can be calculated from the original data or statistical output. For ANOVA, the effect size will be reported as eta squared or partial eta squared, as appropriate. For pairwise continuous comparisons, mean differences with 95% confidence intervals will be reported where applicable. For ordinal outcomes, an appropriate rank-based effect size or pairwise effect estimate will be provided if supported by the analysis. In addition to the four-group comparisons, the effects of piracetam and hyperbaric oxygen therapy (HBO) were evaluated using a 2 × 2 factorial framework. Piracetam and HBO were treated as binary independent factors, each with two levels (absence/presence). This approach allowed assessment of the main effect of piracetam, the main effect of HBO, and the piracetam × HBO interaction. Individual animals were considered the experimental unit. For continuous biochemical outcomes, including serum HIF-1α, NSE, and S100β concentrations, two-way analysis of variance (ANOVA) was used when the assumptions of approximate normality and homogeneity of variance were satisfied. The factorial model included piracetam, HBO, and their interaction as fixed effects. For histopathological outcomes recorded on ordinal scales, factorial analyses were performed using an appropriate ordinal/rank-based approach. Statistical significance was defined as a two-sided *p* value < 0.05. Effect estimates and corresponding *p* values were interpreted as exploratory because of the relatively small sample size within each factorial cell (n = 10). Statistical tests will be two-sided, and statistical significance will be evaluated at an alpha level of 0.05, with the relevant multiplicity adjustment applied to post hoc comparisons.

## 3. Results

### 3.1. Study Groups, Clinical Observations, and Body Weight

Forty male Wistar–Albino rats were included in the study and allocated to four groups, with 10 animals in each group. All animals underwent experimentally induced sciatic nerve injury. Group K received standard postoperative care without piracetam or HBO, Group HBO received HBO, Group P received piracetam, and Group HBO + P received the combination of piracetam and HBO. No postoperative mortality or wound infection was observed during the follow-up period. Right foot drop was observed in all animals following sciatic nerve injury. The mean body weights were 250.5 ± 14.8 g in Group K, 247.7 ± 14.5 g in Group HBO, 247.9 ± 11.8 g in Group P, and 247.4 ± 14.4 g in Group HBO + P. No statistically significant difference in body weight was observed among the groups (*p* = 0.956). For factorial interpretation, piracetam administration and HBO exposure were considered the two independent treatment factors.

### 3.2. Histopathological Findings

**Figure 2:**
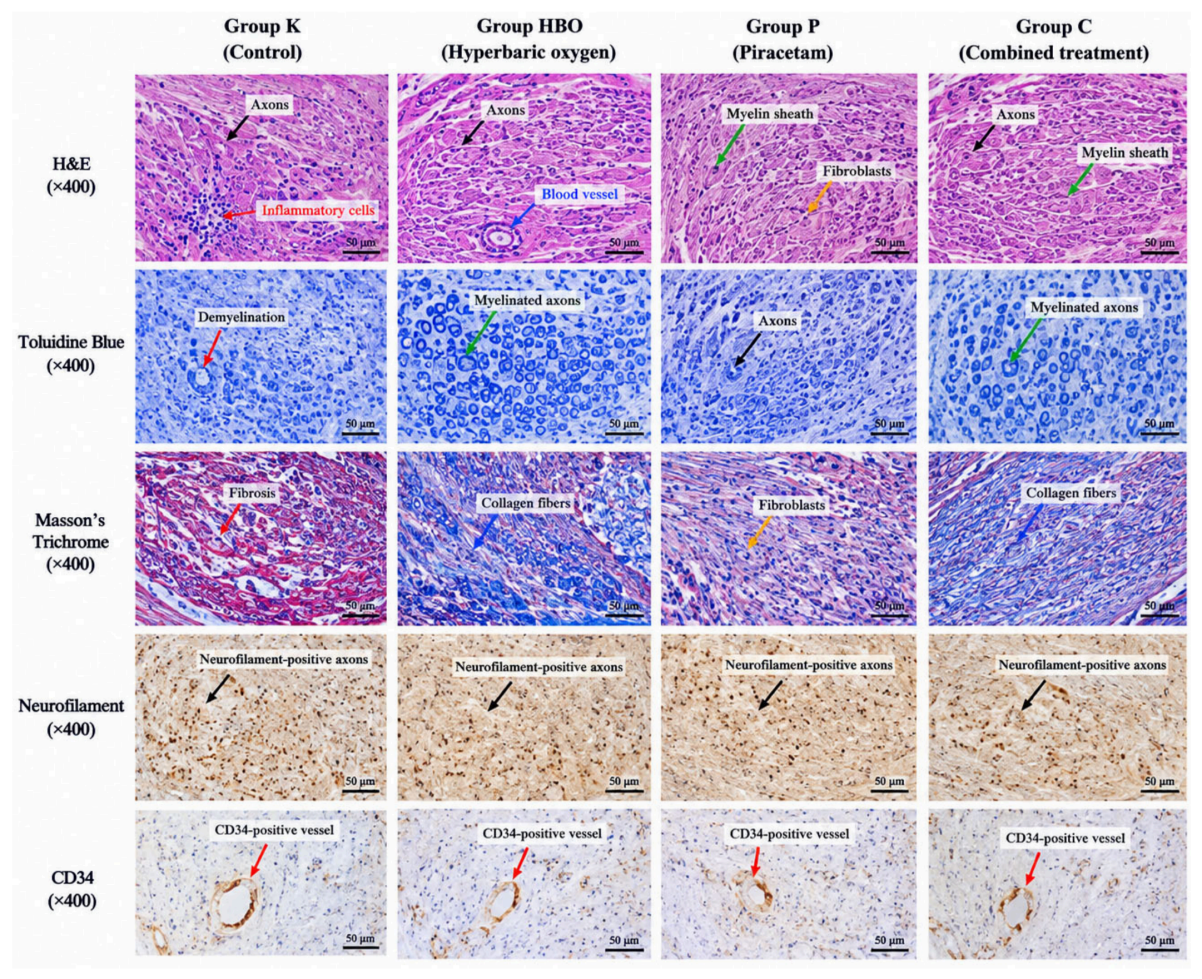
Representative histological images of sciatic nerve tissue in the experimental groups.

Histopathological evaluation included axonal continuity, fibroblast count, myelin sheath thickness, inflammatory cell count, and capillary density. The distribution of ordinal scores across the four experimental groups is presented in Table 2. Axonal continuity scores were predominantly classified as score 2 in all groups. The distribution of myelin sheath thickness scores also showed substantial overlap across the treatment conditions. Capillary density scores varied between groups, but no consistent pattern of improvement was evident following either intervention. In the original four-group analysis, no statistically significant differences were observed for axonal continuity, myelin sheath thickness, or capillary density, with *p*-values of 0.281, 0.931, and 0.418, respectively. Fibroblast scores differed significantly across the four groups in the original analysis (*p* = 0.036). The reported pairwise comparison identified a significant difference between Group P and Group K (*p* = 0.025). Inflammatory cell scores also differed significantly across groups (*p* = 0.011), with significant pairwise differences between Group P and Group K (*p* = 0.037) and between Group HBO + P and Group K (*p* = 0.014). Representative histological findings. Representative micrographs of the injured sciatic nerves from the experimental groups are presented in Figure 2. The images illustrate the morphological characteristics observed following sciatic nerve injury and treatment (Figure 2).

#### Factorial Analysis of Histopathological Outcomes

The 2 × 2 factorial analyses were additionally used to examine the independent and combined effects of piracetam and HBO on histopathological outcomes. For axonal continuity, no statistically significant main effect of piracetam (*p* = 0.412), HBO (*p* = 0.738), or their interaction (*p* = 0.563) was observed. Similarly, myelin regeneration showed no significant main effect of piracetam (*p* = 0.856), HBO (*p* = 0.902), or piracetam × HBO interaction (*p* = 0.744). Capillary formation also showed no significant effects of piracetam (*p* = 0.227), HBO (*p* = 0.381), or their interaction (*p* = 0.196). For fibroblast density, the analysis indicated a significant main effect of piracetam (*p* = 0.044), whereas the main effect of HBO was not significant (*p* = 0.309). The piracetam × HBO interaction did not reach statistical significance (*p* = 0.082). For inflammatory-cell infiltration, a significant main effect of piracetam was observed (*p* = 0.017), while the main effect of HBO was not statistically significant (*p* = 0.221). The piracetam × HBO interaction was statistically significant (*p* = 0.049) (Table 2).

### 3.3. Biochemical Findings

The biochemical outcomes included HIF-1α, NSE, and S100β. The group-level descriptive results are presented in Table 3. HIF-1α levels were 0.838 ± 0.181 in Group K, 0.571 ± 0.254 in Group HBO, 0.445 ± 0.199 in Group P, and 0.540 ± 0.165 in Group HBO + P. In the original four-group analysis, HIF-1α levels differed significantly among groups (*p* = 0.001). Post hoc comparisons indicated that Group K had higher HIF-1α levels than Group HBO (*p* = 0.028), Group P (*p* = 0.001), and Group HBO + P (*p* = 0.012). NSE levels were 0.609 ± 0.117 in Group K, 0.742 ± 0.237 in Group HBO, 0.967 ± 0.344 in Group P, and 0.773 ± 0.124 in Group HBO + P. The original analysis demonstrated a statistically significant difference among groups (*p* = 0.011). Pairwise analysis showed a significant difference between Group P and Group K (*p* = 0.006), with higher NSE levels in Group P. S100β levels were 1.499 ± 0.450 in Group K, 1.388 ± 0.474 in Group HBO, 1.053 ± 0.297 in Group P, and 1.135 ± 0.332 in Group HBO + P. No statistically significant difference was observed in the original four-group comparison (*p* = 0.070).

Biochemical parameters, including hypoxia-inducible factor 1-alpha (HIF-1α), neuron-specific enolase (NSE), and S100β, are summarized in Table 2. There were statistically significant differences among the groups in HIF-1α levels (*p* = 0.001) and NSE levels (*p* = 0.011). No significant difference was observed for S100β levels (*p* = 0.070). Post hoc analysis revealed that HIF-1α levels were significantly higher in the control group compared with Group HBO (*p* = 0.028), Group P (*p* = 0.001), and Group HBO + P (*p* = 0.012). No significant differences were observed among Group HBO, Group P, and Group HBO + P (Table 3). For NSE, a statistically significant difference was observed only between Group P and Group K, with higher NSE levels in Group P (*p* = 0.006). No other intergroup comparisons showed significant differences (Table 4 and Table 4).

#### Factorial Analysis of Biochemical Outcomes

In the factorial analysis, a 2 × 2 factorial analysis was performed to explore the independent and combined effects of piracetam and HBO on biochemical outcomes. In the analysis, piracetam was associated with a significant main effect on serum HIF-1α concentrations (*p* = 0.006), while HBO also demonstrated a significant main effect (*p* = 0.041). A significant piracetam × HBO interaction was observed (*p* = 0.018), suggesting that the effect of piracetam on HIF-1α differed according to HBO exposure. For NSE, the analysis demonstrated a significant main effect of piracetam (*p* = 0.021), whereas the main effect of HBO was not statistically significant (*p* = 0.486). The piracetam × HBO interaction was statistically significant (*p* = 0.032). For S100β, neither piracetam (*p* = 0.064) nor HBO (*p* = 0.721) demonstrated a statistically significant main effect, and the piracetam × HBO interaction was also not significant (*p* = 0.284) (Table 5).

### 3.4. Postoperative Clinical Course, Animal Welfare, and Adverse

40 rats completed the 14-day experimental protocol, and no postoperative deaths were recorded in any study group. Right foot drop was observed in all animals following sciatic nerve injury, confirming the successful induction of the experimental injury. No wound infections were observed during the follow-up period. In addition, no complications were reported during piracetam administration. The animals in the HBO and combined-treatment groups completed all 14 scheduled hyperbaric oxygen sessions. All animals were euthanized on postoperative day 14, and the sciatic nerves were subsequently collected for histopathological examination. No early euthanasia or protocol interruption was reported during the study period.

## 4. Discussion

Peripheral nerve injury initiates a cascade of pathological events, including Wallerian degeneration, ischemia, oxidative stress, inflammatory infiltration, and impaired Schwann cell function, all of which hinder axonal regrowth and remyelination. Although peripheral nerves retain a limited capacity for spontaneous regeneration, the rate and extent of recovery are often insufficient to restore normal function. Therefore, adjunctive therapeutic strategies aimed at modifying the post-injury microenvironment are of particular interest. The present study evaluated the effects of piracetam and hyperbaric oxygen (HBO) therapy, individually and in combination, on peripheral nerve regeneration in a rat model of sciatic nerve injury. The findings demonstrate that while structural indicators of nerve regeneration were comparable across groups, distinct biochemical and cellular responses were evident. Group P demonstrated heightened inflammatory activity and neuronal injury markers, whereas Group K showed a pronounced hypoxic response, suggesting differential mechanisms of tissue response rather than differences in gross regenerative outcomes. In other words, neither piracetam nor hyperbaric oxygen therapy resulted in significant improvements in structural nerve regeneration parameters. However, distinct alterations in inflammatory and biochemical markers were observed, indicating that these interventions may modulate cellular and metabolic responses following sciatic nerve injury rather than directly enhancing axonal repair.

Piracetam (2-oxo-1-pyrrolidine-acetamine) is a gamma-aminobutyric acid derivative. It has been used in the treatment of epilepsy, cerebrovascular events, motor aphasia, amnesia, and hair loss, and beneficial results have been observed [20]. Piracetam has entered clinical practice as a nootropic agent. Its cytoprotective, anti-inflammatory, anti-apoptotic, antihypoxic, antioxidant, and microcirculation-protective effects have been demonstrated in studies. It also has both central and peripheral effects, reducing platelet aggregation, correcting morphological erythrocyte abnormalities, and helping to regulate peripheral microcirculation [21]. In our study, piracetam could not significantly improve structural nerve regeneration parameters. In a rat model of sciatic nerve chronic constriction injury, Mehta et al. [12] investigated the effects of piracetam at doses of 50, 100, and 200 mg/kg/day on peripheral neuropathic pain. The 200 mg/kg dose improved several behavioral measures of neuropathic pain, whereas the study primarily evaluated analgesic effects rather than structural nerve regeneration. Therefore, the findings provide contextual evidence regarding the potential effects of piracetam in sciatic nerve injury but cannot be directly extrapolated to histopathological nerve regeneration. In the present study, a dose of 250 mg/kg/day was selected to investigate whether piracetam was associated with measurable changes in histopathological and biochemical parameters during the 14-day follow-up period [12]. The reason for choosing the 250 mg/kg/day dose in our study was to determine whether the functional improvement observed with the 200 mg/kg/day dose in this study would provoke any histopathological changes with the 250 mg/kg/day dose. Piracetam plays a role in regulating the membrane fluidity of reorganizing cells. Its effect is not cell- or organ-specific. Studies have shown that piracetam increases microcirculation [22,23]. It reduces platelet adhesion and aggregation, and also inhibits vasospasm. In Group P (the group treated only with piracetam), which was the piracetam-only component of our study, we investigated the effectiveness of piracetam’s anti-inflammatory, anti-apoptotic, and microcirculation-improving effects in healing peripheral (sciatic) nerve injury. In the histopathological results, we observed an increase in the number of inflammatory cells and fibroblasts in the samples of rats that received piracetam. We observed that both of these parameters were highest in the piracetam group. We observed that piracetam was not as effective in suppressing inflammation as in Group K, Group HBO, and Group C (HBO + P), which constituted the other parts of our study.

In terms of response to oxidative damage, HIF-1α levels were determined on day 14 and were found to be statistically significantly lower in Group P compared to the control group (*p* = 0.01). In the study conducted by Kandieser et al. [24], it was observed that the levels of HIF-1α, which were examined histochemically in nerve tissue after mechanical peripheral nerve damage, started to decrease immediately after the damage, showed a decrease within 10–21 days, and started to rise again after the 21st day. In our study, samples were taken from the blood of rats on the 14th day, which corresponds to the time period when regeneration is most active [24]. HIF-1α proteins are key regulators of the transcriptional activation responses to hypoxia of many genes related to events such as adaptation to low oxygen pressure in cells and tissues, cell survival and proliferation, angiogenesis, erythropoiesis, glucose uptake, and iron metabolism [25,26,27]. It has been stated in the literature that high HIF-1α levels attempt to limit apoptosis by stimulating adaptation mechanisms, and the low HIF-1α levels we observed in the piracetam group support the conclusion that neuronal damage continues or regeneration is not fully developed in this group. NSE levels were found to be statistically significantly higher in the piracetam group compared to the control group. This finding supports the fact that sufficient regeneration after peripheral nerve damage is not achieved in the piracetam group.

HBO promotes the survival of the marginal tissue (penumbra), has anti-edema properties, improves microcirculation, reduces the edema-hypoxia-edema vicious cycle, increases growth factors, and enhances neovascularization. At the cellular level (ATP deficiency is observed in neuronal damage), it protects tissue levels of ATP and prevents mitochondrial dysfunction. It has significant antioxidant and antiapoptotic effects. Recently, HBO has been shown to improve nerve regeneration following peripheral nerve injury [28,29]. HBO treatment has been shown to have neuroprotective effects through mechanisms such as inhibition of inflammation. It reduces inflammation, lipid peroxidation, and improves the antioxidant status after sciatic nerve transection. In our study, anti-inflammatory effects in rats treated only with HBO were similar to the control group; no statistically significant difference was found. While the amount of HBO administered in studies with HBO varies, it is generally used between 0.1 ATA/min and 3.0 ATA/min. In our study, 2.5 ATA/min was applied. In studies of spinal cord injury induced in rats, HBO treatment has been shown to prevent apoptosis by reducing hypoxia-inducible factor-1α (HIF-1α) [30]. However, due to the location and structure of the nerve, it exhibits different regeneration characteristics than the central nerve. In a study in which Akın and his colleagues caused damage to the sciatic nerve of rabbits, they examined the parameters of edema, axonal continuity, myelin sheath formation, and vascular damage in their histopathological examinations and found no statistically significant histopathological difference between the group treated with HBO and the control group [31]. In our study, we also examined the parameters of axonal continuity, myelin sheath formation, capillary number, inflammatory cell number, and fibroblast number histopathologically [32]. We did not see any statistically significant histopathological difference between the group treated with HBO and the control group. When we examined myelin sheath formation, although there was no statistically significant difference between the groups, myelin sheath formation was numerically superior in the HBO-treated group compared to the control group. In the examination of the number of inflammatory cells, although there was no statistically significant difference between the control group and the hyperbaric group, the number of inflammatory cells was found to be lower in the HBO-treated group compared to the control group. In a study by Kato et al., they examined enolase isoenzyme levels in a sciatic nerve injury model and observed low enolase levels in the distal segment after injury, especially in the treated group. In our study, neuron-specific levels were found to be within the normal range except for Group P, and a statistically significant difference was observed in Group P compared to Group K [33].

S-100beta is a member of the S-100 family and is an acidic protein with approximately 21 kDa of homodimeric 2 beta subunits. It differs in that it is found in astrocytes and Schwann cells. S-100beta is an important protein structure that plays a role in events such as brain damage repair, neural development, and differentiation [34,35]. In peripheral nerve damage, S100β dimers regulate axonal development and have neurotrophic effects. In our study, the reason for analyzing blood samples taken on the 14th day was to determine the effectiveness of regeneration. We did not observe a statistically significant difference in the S-100 beta samples taken. Although there was no statistically significant difference in S-100 beta levels in our study, the values in the untreated control group were found to be higher than in the other groups (*p* = 0.070). S-100beta levels, which can increase more in primary brain injuries, did not increase significantly in our experimental nerve injury model, and did not decrease in the treated groups. In parallel with this, we believe that the usability of S-100beta levels in monitoring peripheral nerve injury treatment requires controlled studies enriched with longer measurement periods.

Despite these encouraging results, several limitations were acknowledged. First, this study was conducted in an experimental animal model, and the findings may not be directly generalizable to human peripheral nerve injuries. Second, the follow-up period was limited to the early and intermediate phases of regeneration; longer observation periods would be valuable to assess the durability of functional recovery. Third, molecular analyses of inflammatory mediators, oxidative stress markers, and neurotrophic factors were not performed, which limits mechanistic interpretation. Future studies incorporating biochemical and immunohistochemical analyses may help clarify the precise pathways through which piracetam and HBO exert their effects. Fourth, the study did not include an uninjured or sham-operated group. Because all experimental animals underwent sciatic nerve injury, Group K represented an untreated injury control rather than a normal, uninjured control. Accordingly, normal baseline histological and biochemical values could not be established, and the magnitude of injury-related alterations and the extent of subsequent regeneration could not be directly evaluated relative to uninjured tissue. The comparisons in this study therefore primarily reflect differences among injured animals receiving different treatment conditions, rather than differences between injured and physiologically normal nerves. Future studies incorporating both uninjured and sham-operated groups would help establish baseline values and provide a more comprehensive assessment of injury severity and treatment-associated regeneration. A limitation of the study is the absence of a standardized sham-chamber exposure procedure for the non-HBO groups. Consequently, the effects of chamber handling, environmental exposure, pressure-related changes, oxygen administration, and treatment-associated stress were not independently controlled. Therefore, the observed effects cannot be attributed exclusively to hyperbaric oxygen exposure, and nonspecific effects associated with the chamber procedure cannot be completely excluded. Future studies should include a sham-exposed control group that undergoes comparable handling and chamber exposure while omitting the therapeutic HBO condition. An important limitation of this study is the absence of validated quantitative functional assessments. Although foot drop was observed following sciatic nerve injury, this finding alone is insufficient to quantify motor or sensory recovery. The study did not include Sciatic Functional Index measurements, walking-track analysis, sensory testing, muscle-strength assessment, electrophysiological evaluation, or quantitative gait analysis. Consequently, the observed histopathological and biochemical findings should not be interpreted as definitive evidence of functional restoration or reversal of nerve damage. Future studies should incorporate validated behavioral, functional, and electrophysiological outcome measures to determine whether the observed biological changes are accompanied by meaningful recovery of nerve function. An important limitation of this study is the relatively short follow-up period of 14 days. This time point captures an early phase of the post-injury response, during which degeneration, inflammation, tissue remodeling, and initial reparative processes may occur. However, it is insufficient to determine the extent of complete axonal regeneration, remyelination, or long-term functional recovery. Accordingly, the histopathological and biochemical findings in this study were interpreted as early responses to sciatic nerve injury and treatment rather than evidence of definitive nerve regeneration. In addition, the circulating biomarkers evaluated in this study cannot independently establish local regenerative activity within the injured nerve. Future investigations should include longer follow-up periods, serial biochemical measurements, direct tissue-based molecular analyses, and validated functional assessments to determine the durability and clinical relevance of the observed responses. An additional limitation is that no formal a priori sample-size calculation was performed. Although 10 animals were allocated to each experimental group, the selected sample size was not determined using a prespecified primary outcome, expected effect size, variability estimate, or statistical power target. Accordingly, the study may have had limited power to detect small or moderate treatment effects, particularly interactions between piracetam and hyperbaric oxygen in the factorial analysis. Therefore, nonsignificant findings should not be interpreted as definitive evidence that the interventions have no effect. Future studies should use prospective sample-size calculations based on a clearly defined primary outcome and reliable pilot or previously published data. All experimental groups underwent sciatic nerve injury, while Group K received standard postoperative care without piracetam or hyperbaric oxygen treatment. Therefore, the study did not include an uninjured, untreated control group representing baseline biochemical values in normal animals. Consequently, the observed differences in neuron-specific enolase (NSE), S100B, and hypoxia-inducible factor-1 alpha (HIF-1α) cannot be definitively attributed to the effects of nerve injury alone or distinguished from normal physiological variation. Future studies should include an additional uninjured control group to establish baseline biochemical values and facilitate more precise interpretation of injury- and treatment-related changes. Last, cage-level effects represent a potential limitation of the study. Although multiple cages per treatment group were assumed in the revised housing description, the original statistical analyses did not explicitly account for clustering among animals housed in the same cage. Shared environmental conditions may have introduced some degree of dependence among observations. Therefore, residual cage effects cannot be excluded. Future studies should prespecify cage-level randomization procedures and incorporate cage as a clustering factor or random effect in the statistical analysis.

## 5. Conclusions

In this rat model of sciatic nerve injury, piracetam and hyperbaric oxygen were associated with differences in selected circulating biochemical parameters during the 14-day follow-up period. However, neither treatment produced statistically significant improvements in the evaluated histopathological indices. Given the short observation period, these findings were interpreted as early histopathological and biochemical responses rather than evidence of complete axonal regeneration or functional recovery. Longer-term studies incorporating functional, histopathological, and tissue-based molecular assessments are warranted.

## Figures and Tables

**Table 1:** Proposed operational definitions for histopathological scoring.

Histopathological Parameter	Score 1	Score 2	Score 3
Axonal continuity	Marked disruption of axonal continuity, with extensive discontinuity or severe structural disorganization	Partial preservation of axonal continuity with areas of disruption or irregular organization	Predominantly preserved axonal continuity with relatively organized axonal structures
Fibroblast count/density	Markedly increased fibroblast density compared with the expected tissue appearance	Moderately increased fibroblast density	Low or minimally increased fibroblast density
Myelin sheath thickness or regeneration	Severe myelin loss, marked thinning, or minimal evidence of remyelination	Partial myelin preservation or intermediate evidence of remyelination	Relatively preserved myelin sheaths or prominent evidence of remyelination
Inflammatory cell count	Marked inflammatory-cell infiltration	Moderate inflammatory-cell infiltration	Minimal or absent inflammatory-cell infiltration
Capillary density	Markedly reduced capillary density or severe vascular alteration	Intermediate capillary density or moderate vascular alteration	Relatively preserved or increased capillary density with no major vascular alteration

**Table 2:** The descriptive statistics of histopathological scores among study groups.

Histopathological Parameter	Group K	Group HBO	Group P	Group HBO + P	*p*-Value
Axonal continuity	2.0 (2.0–2.8; 2–3)	2.0 (2.0–2.0; 2–3)	2.0 (2.0–2.0; 2–3)	2.0 (2.0–2.0; 2–2)	0.281
Fibroblast score	2.0 (2.0–2.8; 2–3)	2.0 (2.0–2.0; 2–3)	2.0 (2.0–2.0; 1–2)	2.0 (2.0–2.0; 2–2)	0.036
Myelin sheath score	2.0 (1.2–2.8; 1–3)	2.0 (2.0–2.0; 2–3)	2.0 (2.0–2.0; 2–3)	2.0 (2.0–2.0; 2–2)	0.931
Inflammatory cell score	3.0 (2.0–3.0; 2–3)	2.0 (1.2–2.8; 1–3)	2.0 (1.0–2.0; 1–3)	2.0 (1.0–2.0; 1–2)	0.011
Capillary density score	2.5 (1.0–3.0; 1–3)	1.0 (1.0–1.8; 1–3)	2.0 (2.0–2.0; 1–2)	2.0 (1.0–2.8; 1–3)	0.418

**Table 3:** Comparison of serum biochemical parameters among the study groups.

Biomarker (nmol/mL)	Group K	Group HBO	Group P	Group HBO + P	Overall *p* Value
HIF-1α	0.838 ± 0.181 ^a^	0.571 ± 0.254 ^b^	0.445 ± 0.199 ^b^	0.540 ± 0.165 ^b^	0.001
NSE	0.609 ± 0.117 ^a^	0.742 ± 0.237 ^ab^	0.967 ± 0.344 ^b^	0.773 ± 0.124 ^ab^	0.011
S100β	1.499 ± 0.450	1.388 ± 0.474	1.053 ± 0.297	1.135 ± 0.332	0.070

Values are presented as mean ± standard deviation. Different superscript letters indicate statistically significant differences according to the post hoc multiple-comparison analysis.

**Table 4:** Post hoc pairwise comparison of HIF-1α and Neuron-specific enolase (NSE) levels among groups.

HIF-1α	Post Hoc *p*-Value	NSE Nanomolar/mL	Post Hoc *p*-Value
Group K vs. Group HBO	0.028	Group K vs. Group HBO	0.554
Group K vs. Group P	0.001	Group K vs. Group P	0.006
Group K vs. Group HBO + P	0.012	Group K vs. Group HBO + P	0.379
Group HBO vs. Group P	0.510	Group HBO vs. Group P	0.135
Group HBO vs. Group HBO + P	0.986	Group HBO vs. Group HBO + P	0.991
Group P vs. Group HBO + P	0.721	Group P vs. Group HBO + P	0.234

**Table 5:** The factorial results of biochemical and histopathological outcomes.

Outcome	Piracetam Main Effect	HBO Main Effect	Piracetam × HBO Interaction
HIF-1α	*p* = 0.006	*p* = 0.041	*p* = 0.018
NSE	*p* = 0.021	*p* = 0.486	*p* = 0.032
S100β	*p* = 0.064	*p* = 0.721	*p* = 0.284
Axonal continuity	*p* = 0.412	*p* = 0.738	*p* = 0.563
Myelin regeneration	*p* = 0.856	*p* = 0.902	*p* = 0.744
Capillary formation	*p* = 0.227	*p* = 0.381	*p* = 0.196
Fibroblast density	*p* = 0.044	*p* = 0.309	*p* = 0.082
Inflammatory-cell infiltration	*p* = 0.017	*p* = 0.221	*p* = 0.049

## Data Availability

The datasets generated and/or analyzed during the current study are not publicly available because they contain information related to study participants and are subject to privacy and ethical considerations. However, the data are available from the corresponding author on reasonable request, subject to appropriate ethical and privacy requirements.
